# Draft Genome Sequence of a Rare Israeli Clinical Isolate of Burkholderia pseudomallei

**DOI:** 10.1128/MRA.00281-19

**Published:** 2019-05-09

**Authors:** Ofir Israeli, Inbar Cohen-Gihon, Tal Brosh-Nissimov, Shay Weiss, Anat Zvi, Adi Beth-Din, Ohad Shifman, Ma’ayan Israeli, Uri Elia, Shirley Lazar, Erez Bar-Haim, Ofer Cohen, Theodor Chitlaru

**Affiliations:** a Department of Biochemistry and Molecular Genetics, Israel Institute for Biological Research, Ness Ziona, Israel; b Infectious Diseases Unit, Assuta Ashdod University Hospital, Ashdod, Israel; c Faculty of Health Sciences, Ben-Gurion University in the Negev, Beersheba, Israel; d Department of Infectious Diseases, Israel Institute for Biological Research, Ness Ziona, Israel; Georgia Institute of Technology

## Abstract

We report here the draft genome sequence of Burkholderia pseudomallei MAA2018. This highly virulent strain was isolated in 2018 from the first melioidosis case in Israel associated with recreational travel to Goa, India.

## ANNOUNCEMENT

The Gram-negative bacterium Burkholderia pseudomallei is the etiological cause of melioidosis, a severe zoonotic infectious disease (165,000 global estimated annual morbidities, including 89,000 deaths [1]) that is endemic mainly in Southeast Asia and northern Australia (2). B. pseudomallei is considered a potential bioweapon (CDC category B) due to the prevalence in soil and water, multiple routes of infection, and especially high virulence associated with inhalation exposure, low infectious dose, high mortality rates, native resistance to a wide range of antibiotics, and nonavailability of a vaccine (3, 4). Melioidosis is manifested by nonspecific symptoms that hinder identification of the disease and consequently may inadvertently be diagnosed as tuberculosis or a common form of pneumonia (1, 5, 6). The B. pseudomallei genome, consisting of two chromosomes, is highly plastic, resulting in a high number of strains exhibiting significant variability in genetic features (7).

We report here the draft genome sequence of a clinical B. pseudomallei strain isolated from a melioidosis case diagnosed in Israel and associated with recreational travel to Goa, India. This is the third documented clinical isolate of melioidosis in Israel (8, 9) and the first case originating from India. The isolate, referred to as B. pseudomallei MAA2018 (melioidosis case from Assuta Hospital, Ashdod, Israel, in 2018) was isolated from sputum collected from a 29-year-old male returning from Goa, India, suffering from a subacute upper-lobe pneumonia of a 6-month duration. The identification of the infectious agent as B. pseudomallei was initially suggested by a metabolism-specific bacterial identification system (Vitek XL2) and matrix-assisted laser desorption ionization–time of flight (MALDI-TOF) spectrum (Vitek mass spectrometer [MS]) and subsequently confirmed by PCR analysis, as described previously (10). Virulence analysis of B. pseudomallei MAA2018 in the murine model of melioidosis established an intranasal 50% lethal dose (LD_50_; determined by following for 30 days the survival of mice inoculated with increasing doses of bacteria and calculated by linear regression using the GraphPad Prism version 5 statistical analysis software [described in references 11 and 12]) of 6 CFU and 30 CFU in the BALB/c and C57BL/6J strains of mice, respectively, suggesting that it belongs to a group of highly virulent B. pseudomallei strains (13).

For the genome sequencing of B. pseudomallei MAA2018, DNA was extracted from a 40-h-old colony grown on Luria broth (LB) agar using the QIAamp DNA blood minikit (Qiagen). A Nextera XT paired-end library (Illumina) was prepared from 1 ng DNA. The library was sequenced on a MiSeq platform (Illumina) using paired-end sequencing with 150 nucleotides (nt) and a mean insertion size of 503 nt. This produced 5,148,334 reads, with 130 coverage (length of the reads × read number/genome length) mapping to B. pseudomallei. For Nanopore sequencing, libraries were prepared from 5 ng of genomic DNA (gDNA) using a rapid PCR barcoding kit (SQK-RPB004; Oxford Nanopore), without fragmentation, and sequenced on the MinION using a MK1 R9.4 flow cell, following the protocol for 1D genomic gDNA, producing 16,809 reads. A hybrid Nanopore-Illumina *de novo* assembly was determined using SPAdes (14), with the following parameters: -t 4 –m 32 –k 31, 51, 71. The number of assembled contigs larger than 1,000 nt was 183. The *N*_50_ contig size was 80,391 nt. FastQC was used to check the raw sequence data for quality control (http://www.bioinformatics.babraham.ac.uk/projects/fastqc/). The draft genome of the MAA2018 strain consisted of 7,423,108 bp (G+C content, 67%). The genomic sequence of the MAA2018 isolate was compared to B. pseudomallei genomes available in the NCBI database, revealing that it differs from its most closely related strain, B. pseudomallei 2008724758 (GenBank accession number CP018382), a clinical isolate collected in 2010 in California from a melioidosis case with unknown travel history (15), by 8,708 and 8,166 single-nucleotide polymorphisms (SNPs) on chromosomes 1 and 2, respectively.

### Data availability.

This whole-genome shotgun project has been deposited at DDBJ/ENA/GenBank under the accession numbers SLUE01000001 to SLUE01000183 (BioProject number PRJNA525961 and BioSample number SAMN11081001). The raw reads were submitted and are available in the Sequence Read Archive at the NCBI as Fast5 files (SRA accession number PRJNA525961).
