# Correction for Israeli et al., “Draft Genome Sequence of a Rare Israeli Clinical Isolate of Burkholderia pseudomallei”

**DOI:** 10.1128/MRA.01105-19

**Published:** 2019-10-03

**Authors:** Ofir Israeli, Inbar Cohen-Gihon, Tal Brosh-Nissimov, Shay Weiss, Anat Zvi, Adi Beth-Din, Ohad Shifman, Ma’ayan Israeli, Uri Elia, Shirley Lazar, Erez Bar-Haim, Ofer Cohen, Theodor Chitlaru

**Affiliations:** a Department of Biochemistry and Molecular Genetics, Israel Institute for Biological Research, Ness Ziona, Israel; b Infectious Diseases Unit, Assuta Ashdod University Hospital, Ashdod, Israel; c Faculty of Health Sciences, Ben-Gurion University in the Negev, Beersheba, Israel; d Department of Infectious Diseases, Israel Institute for Biological Research, Ness Ziona, Israel

## AUTHOR'S CORRECTION

Volume 8, no. 19, e00281-19, 2019, https://doi.org/10.1128/MRA.00281-19. Page 1: The author byline and affiliations should appear as given in this correction.

